# The fate of surplus embryos: ethical and emotional impacts on assisted reproduction

**DOI:** 10.5935/1518-0557.20200015

**Published:** 2020

**Authors:** Cynthia Silva Machado

**Affiliations:** 1 Universidade Estadual Paulista “Julio de Mesquita Filho”- UNESP, Franca, SP, Brazil

**Keywords:** assisted human reproduction, embryo cryopreservation, embryo donation, ethical and emotional aspects, counseling

## Abstract

**Objective:**

This paper looked into the findings of a survey on the ethical and emotional aspects encircling the fate of surplus embryos in Assisted Human Reproduction (AHR).

**Methods:**

Five staff members of a fertility clinic in the Brazilian State of São Paulo answered a semi-structured qualitative interview.

**Results:**

The answers alluded to the different meanings assigned to embryos by medical staff (genetic material) and couples undergoing fertility treatment (potential child). The meaning couples assigned to their embryos, along with inherent uncertainty and distress, affected the choice of what would be done to surplus embryos.

**Conclusion:**

Psychological support may be helpful to two key groups present in assisted human reproduction: clinic staff, for support in their interactions with couples; and couples in need of support and awareness on surplus embryo donation.

## INTRODUCTION

After undergoing treatment with in vitro fertilization (IVF), couples are required by fertility clinics to decide the fate of their surplus embryos (Holwell *et al*., 2014). By signing an informed consent form, they choose between saving the embryos for later cycles, donating them to stem cell research, or donating them to other infertile couples (Fitzgerald *et al*., 2019).

Embryo donation is a procedure associated with many ethical, legal and psychosocial implications. Unlike other countries, Brazil does not have regulation addressing specifically the issues arising from assisted human reproduction (AHR). The only piece of legislation to refer to the matter is Article 5 of Law 11105/2005 (Biosafety Law) on genetically modified organisms promulgated on March 24, 2005, in which the issue of human embryos is discussed (Brazil, 2005; 1988).

Due to this gap in Brazilian legislation, the Federal Board of Medicine (CFM) has debated and issued regulation on the subject (CFM, 2013).

Discussions held at CFM revolve around the ethical norms related to the use AHR techniques and their roles in resolving human reproduction problems and facilitating the procreation process when other therapies were proven ineffective or deemed inadequate (CFM, 2017).

The Brazilian National Health Surveillance Agency (ANVISA) developed a tool called the National Embryo Production System (SisEmbrio) to oversee the production of embryos and analyze data collected from fertility clinics in the nation.

Clinics in Brazil are required to submit data to ANVISA via SisEmbrio on an annual basis and report, among other things, the number of embryos produced, the number of embryos donated for stem cell research, and a wide range of laboratory data used as quality indicators. These reports are used to check for compliance with national standards. According to the 9^th^ National Embryo Production Report (2015), 1,158 embryos have been donated to stem cell research in Brazil since the enactment of the Biosafety Law (ANVISA, 2008). An update issued on February 18, 2016 indicated that SisEmbrio received data from 141 BCTGs or AHR Clinics/Services for embryo production in 2015, revealing that at the time there were 141 registered assisted human reproduction clinics/services in Brazil. Forty-three AHR units were located in the State of São Paulo, the region in which this study was performed. AHR centers failing to submit data to ANVISA are categorized as illegal and subject to notification and penalties as described in Law 6437/1977 (ANVISA, 2016).

Previous studies by Goedeke & Payne (2009) on the theme of embryo donation confirmed the conflict couples experience with donating surplus embryos. Other authors have attempted to seek answers and understand the rationale adopted by couples struggling with embryo donation, the symbolic representations assigned to embryos, and the motivations for donation, among other things. Our study was based on a field survey involving the technical-administrative staff of a clinic in the State of São Paulo, with the purpose of reflecting on the changes in clinical practice and regulation required to provide patient- and healthcare provider-centered care (Lee & Yap, 2003). This study attempted to shed light on the views of technical-administrative staff on the emotional and ethical predicament couples face when deciding what to do with their surplus embryos.

Our study was originally designed to include interviews with couples deciding on the fate of their embryos, but none agreed to join the study. Thus, we had to adjust the study and rely on the witness accounts of staff working at a fertility clinic.

## MATERIAL AND METHODS

We conducted a qualitative survey characterized by the interpretation of interview findings, including weaknesses, potentialities, and the way the interviewees see the matters discussed in the study (Turato, 2003).

The hermeneutic-dialectical method described by Minayo was used in the interpretation of the collected data (Minayo, 2014):

[...] in this method, the speech of the social actors is placed in their context to be better understood. It is an understanding that has at its start the interior of speech and at its end the field of historical and totalizing specificity that produces speech.

### Participants

The study included five members of the technical and administrative staff of a private fertility clinic located in the State of São Paulo. Participants were described in terms of sex, age, and level of education. In terms of sex, 33.3% were males and 66.6% were females. In regard to age, 40% were aged 31-40 years, 20% were aged 41-50 years, 20% were aged 51-60 years, and 20% were aged 61-70 years. All interviewees had higher education degrees.

### Data collection

A semi-structured interview derived from a non-standardized questionnaire was developed for purposes of data collection. The resulting findings were processed and presented in the form of analytical reports.

The questionnaire included questions about the information/explanation provided by clinic staff to couples about the possible fates of cryopreserved embryos, the choices made by the couples from the limited options available, the feelings couples manifested about donating their embryos, the connotative meaning and value assigned to the embryos by couples and clinic staff, and the impressions clinic staff had about the fate of the embryos.

### Procedures

The following steps were taken during the organization of the field survey:

1. A web search was made for private and public fertility clinics in cities near the town of Franca, in the State of São Paulo, where the author took her Master’s Degree.
2. Telephone calls were made to fertility clinics meeting the above criteria.
3. Visits were scheduled with the directors of the clinics willing to join the study.
4. The study design was presented to interested clinics;
5. Interviews with clinic staff were scheduled;
6. Staff members were interviewed with a semi-structured instrument based on a non-standardized questionnaire; interviews were recorded and support notes taken on a field diary.

The interviews were scheduled based on interviewee availability and conducted with one individual at a time at the clinic. The informed consent form was read and signed on the day of the interview.

The following steps were taken after the interviews were over:

1. All empirical field survey findings were listed; recorded interviews were transcribed; interviews were reread; reports and recorded data were organized;
2. Data was categorized and analyzed.

The Research Ethics Committee of the São Paulo State University at Franca (certificate no. 1.319.468) had approved the project that originated this study, with consideration to guidelines for research with human beings, the protection of participant rights, and ethical aspects pointed out in Resolution 196/96 of the National Health Council. Participants were anonymized and the information they provided was treated with confidentiality. They were allowed to withdraw from the study at any time, had access to the author of the study and to study results, and were allowed to use fictitious names during data analysis.

## RESULTS

The interviews made as part of this study were not used in their entirety. A choice was made for answers discussing the possible fates of cryopreserved embryos, the choices made by couples regarding embryo destination, the feelings couples and clinic staff had over the donation of embryos, and the connotative meaning and value assigned to the embryos by the couples and clinic staff.

The first question probed into whether couples knew the possible fates of their surplus embryos and if they were informed of donation options. The couples were reportedly well informed about the process and its developments. At the time of consultation with a physician in the clinic, couples are informed of the treatment to be performed, including the possibility of producing surplus embryos and the options for disposing of cryopreserved embryos. Then they are asked to sign an informed consent form, at which time another clinic staff member repeats the same explanations given before.

Although apparently sufficient, some couples have trouble understanding the meaning of donating surplus embryos to research or to another couple. Individuals with surplus and cryopreserved embryos primarily choose to transfer these embryos to themselves, once the procedure is part of fertility treatment. (Melamed *et al*., 2009). In the words of Cora, one of the interviewees:

They come in and all they want is a baby.

The decision to donate embryos for stem cell research is mostly affected by a couple’s religious beliefs and moral values.

[...] It is quite true that the Brazilian religious field is dominated by the dogmas of Christianity, since we note that Catholicism and Protestantism cover 90% of Brazilians affiliated with some religion in our country. To this vast majority are added other religions and movements that have achieved increasing acceptance and expressiveness. [...]. (Sousa, 2013).

Frieda, another interviewee, shed light on the relevance of religion for some patients:

I had a patient who first came in with a preacher to talk to us and only then came in with her husband.

The second question looked into how interviewees saw the possibility of couples having their surplus embryos transferred to themselves at a later moment, only to find that this was precisely their preference. In the words of interviewee Kahlo:

They say it is part of the treatment, a treatment prerequisite.

Endometrial receptivity may not be at its best at the end of an IVF cycle. Hormone levels may be altered, making the chances of pregnancy decrease to a minimum. In these cases, it might be better to delay the embryo transfer procedure and cryopreserve them for future use.

The third question investigated the impressions derived from donating embryos to infertile couples. This is what Chaplin had to say about it:

In general terms, the donation of a gamete, whether it is an egg or spermatozoa, revolves around giving away something that belongs to an individual. But donating an embryo is a decision that must be made together, by a woman and a man. Some couples ask us if they can leave this decision for later, but we let them know that they must pick an option. And most end up choosing to donate their embryos to research.

Potential donors appear to resist the idea of giving their embryos to other couples.

The fourth question considered the option of donating embryos to stem cell research. Although this is the favorite option for couples seen at the studied fertility clinic, there appears to be a lack of regulation on this mode of embryo donation in Brazil, as Cora described:

The Brazilian Biosafety Law (Brazil, 2005) allows embryos that were frozen until 2005 to be donated for research, while non-viable embryos can be donated at any time.

Chaplin shared his views on what a non-viable embryo is:

These embryos present with anomalies deemed incompatible with life or alterations that impair their development or implantation in the uterus. These can be detected via genetic testing. We have some non-viable embryos here at the clinic.

Chaplin added:

We have the Law from 2005 (Biosafety Law), but ANVISA will soon issue a position statement on the subject. I have been involved in discussions at Pro-Nucleus (Brazilian Association of Embryologists) on the situation of couples donating their embryos for research. Clinics are basically keeping those embryos frozen, since there is no stem cell research going on right now. The University of São Paulo (USP) has received embryos for research, but I am not aware of any institution in which good quality research with design approved by an Ethics Committee is currently in progress.

Another staff member responded emphatically:

In my book this is a beautiful choice, a truly selfless decision. Couples giving their embryos up for research reflect the kind of altruism we need for the progress of science. I must adopt a scientific, technology-oriented attitude in my job. I find it fantastic when couples donate embryos to science, a wonderful thing. This is how new things are discovered. To me, this is why this is essential!

The choice couples make when they sign the informed consent form was the topic of the fifth question. We learned that there is more to it than simply receiving “didactic explanations” about the options in the event couples have surplus embryos. In Kahlo’s words:

This is a complex issue. Couples come in thinking only about their dream children. Cryopreservation is nothing but a vague thought. They do not have surplus embryos to worry about yet.

Guimarães added that the explanation about surplus embryos was delivered at two different times.

I inform the patients during initial consultation and the explanations are given to them again when they are asked to sign the informed consent form.

We learned from interviewing Frieda that patients may also choose to have their embryos preserved indefinitely. In cases of divorce or death of a spouse, this option may be changed. Decision changes must be documented in specific forms. “There is a lot of bureaucracy,” she added.

The choice made in the informed consent form is not final and may be changed if the couple so wishes.

This is what respondents had to say when asked the sixth question, on what these embryos could be:

They are totipotent cells with genetic material from the couple. (Chaplin)

If I had to define surplus embryos in one word, I would call them a shot, a try. I see them as living beings without a life. They are like an unplanted seed. I do not have childish thoughts about them, although that might not be the best word to describe my thoughts about them. I do not have a romanticized view of embryos as frozen living beings in the cold. Some patients ask me about it. To me, they are just cells (Guimarães)

Interviewees recorded their thoughts on the differences between the viewpoints of clinic staff and couples. Clinic personnel are more preponderantly guided by a biological bias, while couples tend to govern their thoughts based on emotions. As described above, couples fear that their embryos might feel cold when they are cryopreserved. Some name their embryos and assign them human traits.

The seventh question addressed the meaning and value assigned to having an embryo. Respondents referred to it simply as the possibility of a future pregnancy.

The eighth question covered what clinics do when the couple decides not to have an embryo transfer. The answer was emphatic:

The clinic keeps the cryopreserved embryos until the couple decides to discard them or donate them to research.

The ninth question wondered whether couples were prejudiced against donating their embryos.

I don't know if “prejudice” is the right word, but I think I can clearly see doubt and fear of making a wrong choice. Very few couples have a less conservative mind [...] The parent-child bond - “this is my son,” “these are my genes,” all of it is quite strong in most cases. (Chaplin)

Chaplin continued:

I think the more we deal with it, talk to couples who cannot have children, watch the advances in science because of donated embryos, the less conservative we become. We are getting more in favor of these alternatives.

Couples have very little information on cryopreservation or embryo donation at the start of treatment or when they are asked to sign an informed consent form.

Although some patients show up more prepared and knowledgeable, many still come in completely clueless.

According to Kahlo,

[...] they come in looking for a child. We go over the process didactically. The technical terminology does not help much, evidently. We also use an illustrated book to explain how it works. But the most important thing is to respect the vision they bring in, respect their feelings, because each couple is unique, has a story, has their own way. There is no such thing as a cake recipe.

Some people say that donating embryos is like donating blood. But it is not. Couples hesitate even though donation is anonymous. They see the embryo as their child. Interestingly, some patients find it laudable to donate embryos for research.

The tenth question looked into whether donating an embryo is an ethical act.

I have no doubt that the word “ethics” fits perfectly with embryo donation, because we follow all the norms and rules of bioethics with regard to AHR. I have no doubt that the donation is ethical and legal. However, I understand that there are lay people, especially individuals who are not from the medical field and who think differently from me, but I do not doubt that it is ethical! (Chaplin)

Other answers:

To me, there is no conflict at all. I agree and accept everything that exists in the AHR Ethical Resolutions, both regarding surplus embryos and homosexual couples, for example. The resolution is well formulated and is consistent with what I think.

Yes, it is an adoption like any other. If you agree to adopt a child, I cannot see why embryo donation might not be ethical.

The eleventh question probed into possible conflicts between one’s personal values and professional practice regarding the fate of surplus embryos. Interviewees had no ethical or emotional conflict with embryo donation practices. They also noted that the resolutions issued by the Federal Board of Medicine and Medical Ethics Committees did a fair job at regulating practice in the area, as stated by Guimarães:

There is no conflict.

The twelfth question referred to what clinic staff believed patients felt about embryo donation. Kahlo described it accurately:

None of our patients has ever expressed a spontaneous desire to donate their embryos to another couple. In some cases, surplus embryos are transferred to the woman in the couple; in other cases, couples choose to donate their embryos for stem cell research.

It is a donation of cells. I see it as giving a chance to a couple that tried and failed to become pregnant. It is about giving them something to hope for. I do not see embryo donation as something inappropriate. It may or not lead to pregnancy.

Chaplin stated:

Few of my patients donated their embryos for research. I felt that they made the donation happily because they knew what they were doing. There was no doubt when they made that decision. Then I realized that they were couples with successful fertility treatments. They were fulfilled couples, their expectations were fulfilled, and their “hearts were full.” I think it becomes easier to think about the success of others when you have already reached your goal. I find it unlikely that couples with failed treatments might donate their embryos.

In Chaplin’s view, achieving pregnancy allows the woman or couple to “fill their hearts with joy” and make the altruistic decision to donate embryos as part of their desire to see others filled with happiness.

The thirteenth question asked participants to share issues they thought should have been included in the questionnaire. In Frieda’s words:

Despite everything we have talked about, in my view embryo donation is not an alternative couples seek. Each couple has their reasons, founded in religion or emotions. I think doctors should merely guide them through the process and ultimately abide by their decision. We are here to support them in their ideas and decisions.

Although the interviews had the specific purpose of capturing thoughts and ideas around the object of the study, additional information and reflections on assisted human reproduction collected during fieldwork were deemed worthy of attention.

The list includes:

- Men's difficulty with handling infertility

Some men tend to confuse infertility with masculinity, although the two are not connected. Therefore, when a man is infertile, the problem gains an additional dimension. Wives tend to be very careful when discussing the issue with their husbands. They do not want to hurt their husbands or have them believe that they expected more from them.

Kahlo added:

When couples facing this situation arrive at the clinic, the male partner is beyond the denial phase of having tests done and seeking treatment. The female partner keeps trying, insisting. Because of this, men take longer to seek treatment. However, by the time they arrive at the clinic, the male partner is willing to undergo treatment.

- Science imposes limits on scientists, as exemplified by Cora:

Science has advanced a lot, but human beings have their limits. There are things we cannot explain. It is possible to keep an embryo inside an incubator for five days. But why will it not develop when it is transferred to a woman’s uterus? We have mastered cell culture techniques, but the intrauterine development of an embryo with mutations is somewhat unpredictable.

The comment above exemplifies the distress experienced by professionals dealing in science and confirms the pain some feel at work:

I try to be very ethical, of course. You might ask me if I ever get too attached. It is not easy. When I am there, I do everything that has to be done. I talk, but that is the way I am. What you have there is just a little cell. But I cannot get too involved, because if I did […] I would probably go crazy. You feel a negative load on your shoulders. This is [...] the experience I have had here in this city. The other day I was in a jeweler’s talking to a store sales rep. The parking lot was behind the store, so you could get in from a back door. This couple came in, and the moment they saw me they turned around and left. Patients do not stay in the sample place as I, because they do not want people to know that they have sought treatment.

Assisted human reproduction is a broad, unprecedented and fantastic area of science. The words of Kahlo allow a glimpse into what the possibility of becoming pregnant might mean for someone who bears the mark of infertility:

Having a child is a very big thing.

Myriad feelings are present in what this patient said, ranging from the narcissistic wound stemmed from the inability procreate to the glory of being able to bear a child thanks to science. Having a child signifies having belief in future generations. It also means that everything required to having a long-awaited child was worth it.

The interviews showed how emblematic embryo donation and all things involved in assisted reproduction are.

It is also clear that embryo donation is an issue to be worked out not only with patients, who need to decide on the fate of their cryopreserved embryos, but with clinic staff living in the boundaries of science.

Bioethics discussions on embryo donation in the realm of assisted reproduction often consider whether surplus embryos deserve protection and if they should be regarded as human beings or mere genetic material, while some wonder whether assisted reproduction has not been used indiscriminately to satisfy the desire of having a child (Silvestre, 2015).

The studies developed by theorists such as Muñoz & Fortes (1998) showed that the relationship that develops in AR - and particularly in embryo donation - involves basically three agents: “[...] the professional, the patient, and society, each with a specific moral meaning: the first guided by the principle of Charity, the second by Autonomy, and the last by Justice.”

This is what we found in our study: (i) the autonomy and interest of patients deciding on the future of their cryopreserved surplus embryos; (ii) the professionals treating the patients, mindful of patient autonomy in deciding the fate of embryos and who as a rule prevent and do not cause harm to the patients and safeguard their wellbeing; (iii) society, the eventual beneficiaries of stem cell research, and couples with a desire to become parents benefitting from embryo donation (Féo, 2010).

We emphasize that the embryo is a vulnerable subject and cannot be considered as a means. Although couples are covered in some of the pillars that govern bioethics - information, understanding, and willingness - the interviews revealed that information alone does not guarantee couple decision-making autonomy (Mandelbaum *et al*., 1998). The affective bond couples develop toward their embryos appeared in the interviews. Although couples are provided with “didactic explanations” concerning the options they have for their surplus embryos, the expectations related to having a child and fulfilling parenting desires affect the way couples understand the process, which also includes the possibility of oocyte overproduction and cryopreservation of embryos. In their minds, cryopreservation is only a distant reality, not something they should be concerned with yet. Therefore, deciding on the fate of surplus embryos becomes an even more distant idea (Silva *et al*., 2017).

Another pattern seen in the interviews is the preference for postponing the decision of what to do with surplus embryos. This is an issue to be discussed with couples, since the literature has shown that postponing the decision often leads to embryos being left indefinitely in assisted reproduction clinics (Perelson & Haski, 2015). Abandoning embryos in clinics is not uncommon after couples have seen the dream of having a child come true, as they detach themselves from the responsibility of making a decision about their surplus embryos (Perelson, 2009).

## DISCUSSION

The views reported in our study showed that the bond couples develop with their embryos and the other two explanations offered by clinic staff do not seem to be enough to fully explain couple decision-making. Couples undergoing fertility treatment experience significant emotional distress. The expectations and anxiety associated with getting pregnant are obviously projected on surplus embryos. We might say from the interviews that couples establish a bond with their embryos that goes beyond what an embryo means in scientific terminology. The connotative link between an embryo and a child might explain the difficulty couples have with donating embryos.

Previous studies showed that couples hesitate to decide whether or not to continue to store their frozen embryos. Several aspects influence a couple’s decision, including the meaning they attach to their embryos, information and support provided by the fertility clinic, the quality of the embryos, and other circumstances (Bruno *et al*., 2016). Although couples are offered information on embryo donation, the feelings inherent to the process - doubt, anxiety, expectation, fear, prejudice - must be properly considered. The willingness of clinic staff alone to provide additional information might not be sufficient to resolve the conflicts tied to making a decision about surplus embryos (Raz *et al*., 2016).

Embryo donation deserves more careful analysis with consideration to items such as the feelings, doubt, desires, and expectations of couples.

Couples should face embryo donation with rationality. The meaning couples assign to their embryos as a result of what they have experienced in life cannot be ignored. Some findings did not come out strictly from the answers participants gave to the questions, but from the impressions they gathered from interacting with patients. These included the feelings they had toward patients, the limits of science, and how much these factors affect the lives of clinic staff. Counseling sessions with a psychologist may help patients to work out their emotional issues and build awareness over the importance of embryo donation. Individuals working at fertility clinics faced with ethical and emotional challenges in a daily basis - feelings of helplessness, anxiety, and distress - may also benefit from psychological support.
